# The Hospital World

**Published:** 1911-08-19

**Authors:** 


### The Hospital World.

### A Bournemouth Doctor's Estate.

Dr. James Paul McGeagh, of Bradstone, Branksome Park, Bournemouth, who died on February 17, has left estate valued at £56,806 gross and £54,992 net.

### A Local Supporter of Addenbrooke's.

The Governors of Addenbrooke's Hospital, Cambridge, have recently received £100 from Mr. Chas. Finch Foster, J.P., D.L., of Cambridge, as a special donation. Mr. Finch Foster is an annual subscriber of £25, and in addition has given £500 to the general funds during the past few years and £1,000 to the King Edward VII. Memorial Fund.

### Coronation Festivities at Leicester.

The subscribers to the Coronation Festivities Fund at Leicester report a balance in hand of approximately £750 after all expenses have been paid. At Mr. W. Vincent, the Mayor's, suggestion, £250 of this will be devoted to the Leicester Provident Dispensary Hospital, £250 to the District Nurses' Association, and the balance to the Leicester Infirmary and Children's Hospital.

### The New Hospital at Antrain, France.

We have received news of the inauguration of the new hospital at Antrain at which M. Messimy, Minister of War, and M. Etienne, Vice-President of the Chamber, presided. They were received by MM. Le Hérissé, Surcouf, Guernier, and Lefas, deputies; Dr. Labbé, senator of l'Orne; M. Saint, préfect; General Lyautey; M. Garreau, a former senator; M. Janvier, Mayor of Rennes. M. Etienne, whose speech was much applauded, paid homage to the army and its chiefs, "Who," he said, "whatever may happen, will know how to do their duty, and are equal to the trust that the country has put in them."

### A Loss to the Hanwell Cottage Hospital Staff.

Mr. Alfred Derwent Maitland, a well-known practitioner of Hanwell, died on August 11 at a nursing home. He was a student at University College Hospital, and became qualified in 1881 as M.R.C.S. Eng., taking the L.S.A. in the following year and the L.R.C.P. in 1884. He did a great deal of careful work for the Hanwell Cottage Hospital, to which he was honorary surgeon. His earlier experience was obtained in surgical appointments at the Sunderland Infirmary, the Scarborough and Hartlepool Hospitals, and as clinical assistant at the Soho Hospital for Women.

### An Irish Coroner and Rifle Shot.

The death is announced of Dr. John Christopher Sellars, of Dundalk, co. Louth, a prominent member of the profession in Ireland. He held the offices of Coroner and Medical Officer of Health for Dundalk, and was a noted performer at the rifle ranges. He was a familiar figure at the meetings of the National Rifle Association held at Bisley and Wimbledon, and for several years was the Irish champion. He frequently shot in the Irish Eight for the Elcho Shield, and was a winner of the Lord Lieutenant's prize. He qualified in 1884 at the Royal College of Surgeons of Ireland, after having studied at the Catholic University, Dublin. He also held the L.M. of the Rotunda Hospital and the Licentiate of the Royal College of Physicians of Ireland.

### Death of a Mutiny Medical Officer.

The death is announced of Deputy Surgeon-General John Jones, at the age of eighty-one. He qualified in 1853 and became a member of the Royal College of Physicians in 1860. The University of St. Andrews claimed him as M.D. in 1865. At an early stage of his career he served in the Indian Mutiny, and was present at the operations in Oude and at the actions of Naruone, Karkarala, and Mehndee. He received the Mutiny medal, and has been on the retired list of the Indian Medical Service since 1885. His death took place last week at his house in Cromwell Road, and he was buried at Brompton Cemetery.

### Death of Dr. Samuel Gee.

St. Bartholomew's Hospital has just lost one of its most eminent physicians in the person of Dr. Samuel Jones Gee. His well-known book of "Medical Lectures and Aphorisms" attained a great popularity on account of its ingenious and entertaining thoughts in which important medical truths were compressed into brisk, didactic paragraphs. As a physician he will always be remembered by a work on "Auscultation and Percussion," which has run into many editions, and still remains one of our most valuable text-books. His contributions to medical literature were very numerous, and he gave in succession the Galstonian lectures in 1871, the Bradshaw lectures on acute peritoneal diseases in 1892, and the Lumleian lectures on bronchitis, emphysema, and asthma in 1899. Dr. Gee has been associated with the Royal College of Physicians for close on half a century, taking his membership about the year 1866 and becoming a Fellow in 1870. He held the office of Censor three times, the last being in 1897. At Bart's he was always a great favourite with the students, and had a large following in the wards. It was a matter of both practical and sentimental regret when, in due course, this great clinician had partially to retire and become a consulting physician to the hospital.

### Hospitals in Armenia To-day.

A Correspondent writes: It is a far cry from London hospitals to the buildings which have to do duty in Armenia. The suddenness with which persecution time after time has overtaken Christians there has not allowed much progress. The first hospital at Adana came suddenly into existence. On April 14, 1910, Major Doughty-Wylie and his wife heard that "something was happening at Adana." On their arrival an Armenian was killed in front of them, and whilst the Major rode off to stop the riot, Mrs. Doughty-Wylie soon found an impromptu hospital on her hands. In the guard-house opposite the Consulate the wounded people had been left. On Sunday, April 25, a second massacre brought sixty-nine in-patients and 250 out-patients. Food was scarce and so dear that the little there was seemed out of reach. The provision of a second house as hospital gave sixty more beds to the work, the former building being used for medical work. The emergency hospitals thus established have now been merged into one central building. A gift of £1,000 offered by the International Relief Committee towards founding a permanent hospital ensured the first year's expenses, and "the Famine Building" was opened up and fitted out as a permanent hospital. In February of this year statistics showed a daily attendance at the dispensary of 7,700 for the year, 2,000 of these being Moslems; while 1,200 visits were paid by the doctor. The in-patient department totalled

301 patients, ninety-five being women. A letter from Miss Wallis, who has been working in Adana since 1903, says: "Our hospital is advancing in the upward grade of civilisation; the bedsteads have come, and all the old beds on the floor are removed. What has been done at Adana is also being carried on at Sivas and Van. The poverty of the district makes illness general, and when some disease such as typhus swoops down, practically no resistance is offered. As far as possible the dispensary work is carried on on self-supporting lines, the patients in one year having paid half of the cost of their dressings and treatments, and the hospital patients 60 per cent. of the amount spent on them. The actual cost of each patient is not great, one shilling and eightpence per day, or £24 a year. Van Hospital was built with the intention of accommodating fifty patients, but sometimes seventy are crowded in. Last winter was the coldest for forty years, and the work in the wards is made difficult by reason of the poor supply of blankets and hot-water bottles. There is no room set aside for drying-laundry, and the difficulty is to keep the patients clean and yet avoid pneumonia, through making use of garments insufficiently aired.

